# Motives Behind Dairy Product Purchasing Decisions Among Polish Doctors: An Age-Based Analysis

**DOI:** 10.3390/foods14183169

**Published:** 2025-09-11

**Authors:** Anna Goliszek, Sebastian Białoskurski, Agnieszka Komor, Anna Nowak, Aneta Jarosz-Angowska, Artur Krukowski, Katarzyna E. Przybyłowicz, Katarzyna Staniewska, Aneta Dąbrowska

**Affiliations:** 1 Department of Management and Marketing, Faculty of Agrobioengineering, University of Life Sciences in Lublin, Akademicka 13, 20-950 Lublin, Poland; anna.goliszek@up.lublin.pl (A.G.); agnieszka.komor@up.lublin.pl (A.K.); 2 Department of Economics and Agribusiness, Faculty of Agrobioengineering, University of Life Sciences in Lublin, Akademicka 13, 20-950 Lublin, Poland; anna.nowak@up.lublin.pl (A.N.); aneta.angowska@up.lublin.pl (A.J.-A.); artur.krukowski@up.lublin.pl (A.K.); 3 Department of Human Nutrition, Faculty of Food Sciences, University of Warmia and Mazury in Olsztyn, Słoneczna 45f, 10-718 Olsztyn, Poland; katarzyna.przybylowicz@uwm.edu.pl; 4 Department of Commodity Science and Food Analysis, Faculty of Food Science, University of Warmia and Mazury in Olsztyn, Pl. Cieszyński 1, 10-726 Olsztyn, Poland; kasta@uwm.edu.pl; 5 Department of Dairy Science and Quality Management, Faculty of Food Science, University of Warmia and Mazury in Olsztyn, Oczapowskiego 7/8, 10-719 Olsztyn, Poland; anetazj@uwm.edu.pl

**Keywords:** dairy products, doctors’ purchasing decisions, buyers’ segmentation, age-based analysis

## Abstract

The aim of this article was to identify factors influencing the purchasing decisions of individual buyers represented by Polish doctors on the dairy product market, taking into account the demographic variable of the respondents’ age. This article is based on a survey conducted among 201 Polish doctors using the CAPI (Computer-Assisted Personal Interview) method. The collected primary data was subjected to quantitative analysis, including both a description of general trends and the identification of differences in responses between distinct age categories of respondents, as well as the identification of hidden factor structures. Conducting a study with a group of doctors perceived as experts fills a research gap in the literature and makes an important contribution to the discussion on the factors shaping the purchasing behaviour of doctors as buyers. The research revealed differences in the assessment of the importance of motivators influencing purchasing decisions for dairy products between groups of doctors separated by age. Younger respondents (aged 27–44) attach greater importance to the influence of marketing activities at the point of sale and the health attributes of dairy products, while older respondents (aged 45 and over) attach greater importance to aspects related to trust and safety as well as consumer trends. Hidden (latent) factors influencing purchasing decisions in the surveyed age-based groups of doctors were also identified in comparison with the total number of respondents, and consumer segments were identified based on the similarity of factor profiles. The results of this study can be used both by companies in the dairy sector in the process of designing marketing strategies for dairy products and by institutions influencing public health.

## 1. Introduction

The literature emphasises that consumer purchasing behaviour is the result of a complex combination of factors. Dairy products, due to their diversity and important role in the daily diet, constitute a particularly interesting category for analysing consumption motives. In this context, identifying the key determinants of purchasing decisions becomes crucial for marketing practices and competitive strategies of companies. Purchasing motives for this product group can vary significantly depending on the consumer group, identified based on selected characteristics, including demographic data. Understanding the mechanisms behind the decision-making process provides the foundation for effectively competing in the dairy product market. This stems from the fact that, according to the marketing concept, in order to compete effectively in the market, product suppliers should focus their activities on final buyers, giving priority to their individual expectations and needs. The traditional marketing approach is based on satisfying the needs of customers representing the company’s target market [1,2,3]. Currently, due to the social and environmental challenges facing the modern world [4], in addition to focusing on the needs of final buyers, companies’ marketing activities also pay attention to issues of sustainable production and consumption [5,6,7]. This requires manufacturers to take into account aspects related to environmental protection, broadly understood food quality and proper nutrition of consumers in the process of creating their product offer [8,9]. These aspects are present in many contemporary concepts of market activity, such as eco-marketing, social orientation and sustainable development [10,11], as well as in some contemporary consumer trends, such as sustainable consumption [6,12]. These orientations and trends contribute to the promotion of healthy eating, proper consumption habits and purchasing behaviour, which in turn affect the quality of life, health and well-being of consumers and entire societies. When creating products, food product suppliers should consider the marketing aspect, ensuring that potential buyers will be willing to purchase them at a time and place that is convenient for them and at an acceptable price [13]. This approach is particularly important when there is a greater likelihood of better market offers, when products are more interchangeable and when the market is more saturated. Therefore, it can be concluded that this approach is particularly important in the food market, including the dairy market [8].

On the other hand, marketing activities should be linked to the concept of sustainable development in order to strive for human well-being without exposing future generations to various dangers [14]. It is also important to offer valuable products, including functional products, which allow consumers to take care of their own health and well-being and that of other members of their household [15,16,17]. Consumers, especially those who are environmentally and health conscious, are increasingly seeking information about the nutritional properties and health effects of a given product. Therefore, understanding consumer behaviour in terms of food consumption, especially with regard to health aspects, is essential for promoting sustainable and healthy food choices [18].

Nowadays, effective marketing of food products, including the commercialisation of new products, should be based on an analysis of current and potential needs (especially in the case of so-called new products), but also on the identification of factors determining the purchasing behaviour of buyers [19]. Despite the fact that analysing consumer purchasing behaviour and drawing appropriate conclusions that can be applied in the marketing strategies of food product suppliers is very difficult, multidimensional and complex, it is necessary to understand consumer behaviour and the motives that guide them when making purchasing decisions [8,20,21]. Furthermore, due to the diversity of buyers’ purchasing behaviour and its determinants, an important aspect of a company’s marketing orientation is market segmentation, which allows for the identification of homogeneous groups of customers who define their area of operation and form the basis for the development of marketing strategies [22,23,24].

Various factors influence buyers’ purchasing reactions [25,26]. These include needs, motives, perceptions, attitudes, personality, learning and risk-taking, as well as social, cultural, situational and economic conditions [3,27]. The demographic and economic profile of the customer, which includes variables such as age, gender, place of residence, family status, income, occupation and level of education, is also noteworthy. These factors are taken into account in the customer segmentation process [27,28], as they allow us to answer the question of who constitutes the customer base of a given supplier and why they purchase certain products. This is particularly important in relation to food products, where purchasing motives change dynamically. This is due to the fact that food is consumed in specific places, in a specific social context (e.g., alone or in the company of others) and at different times of the day [29]. In addition, research shows that demographic criteria shape the purchasing decisions of food buyers [30]. Due to their ease of measurement, they are widely used in market segmentation practice. In addition, demographic criteria are communicative and unambiguous, as well as generally available. Commonly used variables include age, gender, race, religion, nationality, education, occupation and social status. Age is a particularly frequently used segmentation variable, as it usually determines other variables such as education level, family structure and income [30,31,32]. This led the authors of this study to use it in their research on the purchasing behaviour of doctors who buy dairy products.

In addition to demographic criteria, psychographic and behavioural criteria are also used in the segmentation process [33]. This group includes variables related to the buyer’s activity (professional work, hobbies, culture, entertainment, sport, etc.) and interests (family, home, job, neighbourhood, fashion, nutrition, etc.) and variables that take into account lifestyle, which refer to the scope and forms of everyday behaviour specific to a particular social group or individual, i.e., a characteristic way of behaving in society. Currently, in the context of food products available on the market, consumer interest in a so-called healthy lifestyle and sustainable consumption plays a huge role [3,12,34,35,36]. Recent studies indicate that behavioural segmentation based on consumer purchasing patterns and preferences is gaining importance [37]. By understanding the determinants of purchasing decisions, food producers find it easier to create new product offers, position them appropriately and develop effective promotional strategies [38,39].

A review of the literature reveals a research gap in identifying the factors influencing the purchasing decisions of dairy product consumers, especially with regard to their age. Studies on the segmentation of consumers of this product group are also rare. The available research often focuses only on limited segmentation criteria or on selected products. For example, Yue et al. [40] assessed the psychological factors determining consumer attitudes towards sustainably produced meat and dairy products in five European countries (the Czech Republic, Spain, Sweden, Switzerland and the UK). They also segmented the market based on consumers’ attitudes towards sustainable food, sensory and health characteristics. Meyerding and Seidemann [41] analysed how different characteristics of milk—such as packaging, farming practices, feeding methods, price and price transparency—influence consumer decisions in Germany. However, their study was limited to fresh milk as a case study. Sajdakowska et al. [42] also referred to dairy products in their study, assessing consumers’ attitudes towards quality and identifying five consumer clusters. However, this study was limited to quality aspects and covered a broader group of animal-based foods, including dairy products.

This study attempts to fill this research gap by identifying factors influencing the purchasing decisions of individual buyers representing the Polish healthcare sector in the dairy product market, taking into account the demographic variable of the respondents’ age. An equally important objective of this study was to segment respondents with similar factor profiles, i.e., similar response patterns, within the total sample and within the selected age categories. This study analysed the influence of age on purchasing decisions of Polish doctors, a professional group with high opinion-forming potential in the field of nutrition and health. The inclusion of the variable of the age of the respondent doctors in this study facilitates the identification of differences in preferences and purchasing behaviour, which may be helpful in market segmentation, the designation of marketing campaigns for dairy products or the adaptation of the product range to different target groups. Due to the specific nature of the respondent group, which influences the dietary attitudes of various social groups, the results of this study can be used by companies in the dairy sector in the process of developing their marketing strategies, including the launch of new products on the market. Furthermore, the results of this research may provide important guidance for decision-makers in institutions influencing public health by creating campaigns aimed at promoting healthy eating habits that include the consumption of dairy products as part of a balanced diet beneficial to public health.

## 2. Materials and Methods

In the course of the research, the following research hypotheses were formulated and then verified:

**H1.**  *There are differences in the assessment of the importance of motivators influencing purchasing decisions for dairy products between surveyed groups of doctors separated by age (≤44 years and ≥45 years), which is reflected both in the hierarchical arrangement and in the strength of the assessments of individual variables.*

**H2.**  *There are differences in the structure of hidden (latent) factors influencing purchasing decisions in the dairy product market depending on the age category of respondents, which reflects different surveyed buying doctors’ profiles.*

In order to achieve the objective of this article and verify the research hypotheses, empirical research was conducted. The primary data collection method was a survey. The research was conducted using the CAPI (Computer-Assisted Personal Interview) technique among 201 final buyers representing the health service in Poland. Admittedly, the healthcare sector is multi-layered and also includes other professional groups that can influence consumer decisions. However, in this study, we focused on doctors because they play a special role in shaping public opinion and enjoy high social authority in the field of health and nutrition. The choice of this group of respondents was therefore justified by the purpose of the analysis—we wanted to capture the attitudes and recommendations of people whose opinions have the greatest influence on shaping the eating habits of society. In Poland, in 2023, the number of doctors working directly with patients was 141,500. Women predominate in the doctor population—59%. In that year, the average age for men was 48 years, and for women, 50 years. The sample was selected using a purposive sampling method. The criteria for participation in the study were being a practising doctor and having direct contact with patients. The survey was conducted nationwide, covering all Polish provinces at the EU NUTS 2 level (there are 16 NUTS 2 regions in Poland). Respondents were selected in proportion to the population of each province, which means that proportional territorial allocation was used. This study included all provincial capitals, and the established quotas were supplemented with respondents from randomly selected smaller cities and towns. Although not random, the sample selection strategy we used ensures the spatial diversity of the respondents and increases the geographical representativeness of the study results.

This article covers an independent variable—the age of respondents—and a dependent variable—factors influencing purchasing decisions on the dairy market. As part of this study, respondents were asked to enter their year of birth (open question), on the basis of which their age was determined. The respondents were divided into two age categories based on assumptions related to the stages of professional development and the specific nature of doctors’ career paths: doctors up to 44 years of age and doctors over 45 years of age. In the survey, respondents were also asked to assess the factors influencing their purchasing decisions in the dairy products market. For this purpose, respondents were presented with a set of 26 variables identified on the basis of a cognitive–critical analysis of the literature [43,44,45] and based on the data obtained using the Delphi method. As part of this procedure, a team of six experts specialising in consumer behaviour was asked to identify and organise the key purchasing motives relevant from the perspective of the dairy product market. Each element of the set selected through the above procedure was then assessed by respondents in terms of its importance in purchasing decisions on a 5-point Likert scale, which is one of the most basic and widely used psychometric tools in social sciences [46].

The collected primary data was subjected to quantitative analysis, including both a description of general trends and the identification of differences in responses between distinct age categories of respondents, as well as the identification of hidden factor structures. Central tendency measures (arithmetic mean) were used for statistical analysis [47], which, using a ranking procedure [48], were used to determine the hierarchy of factors influencing purchasing decisions in the dairy product market for all respondents and for both age groups of doctors. The statistical description was supplemented with measures of dispersion (standard deviation, coefficient of variation), enabling the assessment of the diversity of responses in the surveyed categories of respondents [49]. In order to identify differences in the selected groups of respondents, a comparative analysis method was used, as well as the non-parametric Mann–Whitney U test, which is appropriate when the data does not meet the assumptions of parametric tests and when the dependent variable is measured at least on an ordinal scale [50]. In addition, an exploratory factor analysis using the principal component method was conducted to identify hidden structures of factors influencing purchasing decisions. It allowed the transformation of a set of correlated variables into a set of hidden variables (factors/principal components), explaining the interrelationships between observations and reaching deeper structures of the studied reality [51]. The analysis was conducted separately for all respondents and for groups separated by age. The Kaiser criterion was used to determine the number of factors, assuming that only factors with eigenvalues greater than 1 were taken into account [52]. The factors were rotated using the Varimax method, while a minimum factor loading value of 0.5 was adopted for the interpretation of the factor structure, which is a margin often used in the literature [53,54]. Statistical analysis of the collected primary data was performed using PS Imago Pro Academic, version 10.

## 3. Results

This chapter presents the results of empirical research on factors influencing the purchasing decisions of doctors as consumers of dairy products. The survey was conducted on a sample of 201 Polish doctors. Among the respondents, there were 120 women (59.7%) and 81 men (40.3%). The gender structure of the respondents reflects the general trends observed in the population of doctors in Poland, where women account for approximately 59% of the total. The survey participants represented the following age groups: 25–34 years old—14.9%; 35–44 years old—31.3%; 45–54 years old—30.3%; 55 years old or older—23.4%. For the purposes of the analysis, respondents were divided into two age groups. The first group consisted of younger doctors, up to 44 years of age (46%, or 93 individuals), who were in the process of shaping and developing their career path. The second group consisted of doctors aged 45 and over (54%, or 108 individuals), whose professional situation could be described as stable. The doctors we surveyed represented 23 different medical specialisations, the most frequently indicated of which were internist—75 responses—and paediatrics—57 responses. The vast majority of respondents worked in cities (92%), while 8% were employed in rural areas. Due to the deliberate selection of the sample, the study was not representative, and the results can only be applied to the population we surveyed.

**Figure 1:**
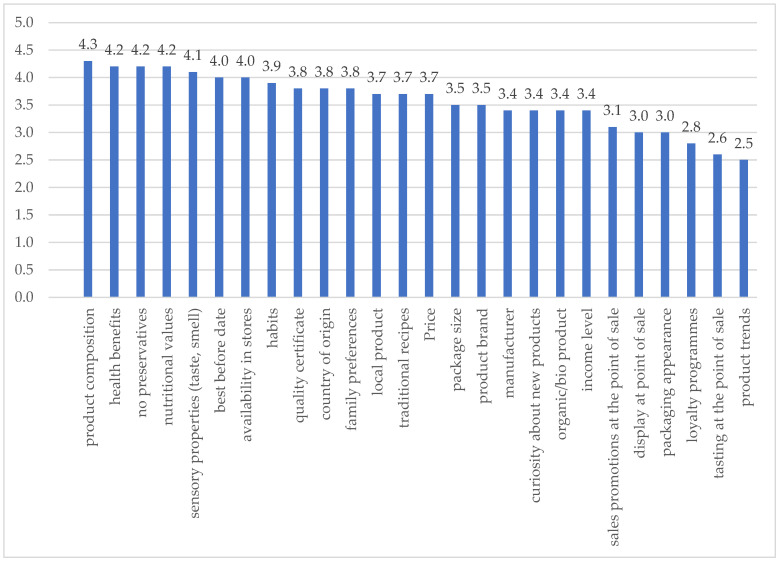
Arithmetic mean of the importance of factors influencing purchasing decisions for dairy products: 1—completely unimportant, 2—not very important, 3—difficult to say, 4—rather important, 5—very important. Source: own research results.

In order to achieve the research objective, respondents—who were purchasers of dairy products and representatives of the medical community—were presented with a set of variables that could potentially influence their purchasing decisions, which concerned the following (Figure 1):

- The health benefits of dairy products;
- Quality attributes (including product safety);
- The product’s provenance and trustworthiness;
- Economic and practical qualities;
- Qualities related to marketing factors (including those related to impact at the point of sale);
- Social and psychological issues (including habit, family preferences or curiosity about a new product).

The average rating of individual variables ranged from 2.5 to 4.3, which indicates a varied level of their importance in the purchasing decision-making process of the surveyed doctors on the dairy product market. The highest average rating of the purchasing motives for dairy products in the analysed group of doctors in Poland was given to health-related and quality factors (product composition, health benefits, absence of preservatives, nutritional value, sensory properties, shelf life, availability in stores). Slightly less important in the respondents’ opinion were psychosocial factors related to eating habits (habits, preferences of family members), food safety, product origin (country of origin, local products, traditional recipes, product brand) as well as economic and practical aspects (price, package size). Consumer choices are also moderately influenced by communication activities that create the image of a product as a “market novelty” and “organic product”, marketing activities (sales promotions at the point of sale, display at the point of sale, packaging appearance, manufacturer) and the consumer’s financial situation. According to the responding doctors, loyalty programmes, tastings at the point of sale and product trends are the least effective motivators for purchasing dairy products.

### 3.1. Motives for Purchasing Dairy Products and the Age of Respondents

In order to verify H1, which assumes that there are differences in the assessment of the importance of motivators influencing purchasing decisions for dairy products between doctors aged up to 44 and doctors over 45, this article compares the ratings given to individual motivators in distinct age categories of doctors. The aim of the comparison was to identify possible differences in the hierarchy of importance of individual motivations influencing purchasing decisions in the opinion of separate age categories of doctors and to show differences in the strength of assessments of individual motivators.

The arithmetic mean for purchasing decision motives in the dairy product market, depending on the age of the surveyed doctors, ranges from 2.4 to 4.3 for younger people (i.e., aged 27–44) and between 2.7 and 4.3 for respondents in older age groups (i.e., aged 45 and over). Higher minimum ratings are therefore found in the older group of respondents (Table 1), which may suggest that greater importance is attached by them to the analysed motivators. In both groups of respondents, the composition of the product was of the greatest importance—this variable obtained the highest average value among both younger and older respondents (in both cases, it was 4.29) as well as the highest percentage of declarations that it is a very important or important factor in the decision-making process (this was indicated by nearly 80% of younger respondents and over 84% of older respondents). In addition, the basic motives declared to influence purchasing decisions regarding dairy products among younger respondents are, in order, health and nutritional values as well as the absence of preservatives and sensory properties; in the case of older respondents, they are the absence of preservatives, sensory properties, shelf life, health and nutritional value. For these variables, the average scores were at least 4.0. Each motive in this group is considered an important or very important factor in the decision-making process by at least 75.3% of younger doctors and 76.9% of older doctors. These results indicate that although the most important purchasing motives in both groups are factors indicating health-promoting and quality motivations, with product composition at the top, their structure differs in terms of the emphasised motivators. For younger doctors, health aspects are prioritised, whereas older doctors tend to emphasise more pragmatic and sensory aspects. The second group of motives influencing the purchasing decisions of younger and older respondents with regard to dairy products are those with an average score of 3.5–3.9. This group includes food safety aspects (quality certificate), product origin (country of origin, local product, traditional recipes), economic and practical aspects (price, package size, availability in stores) and eating habits (habits, preferences of family members). These motivations are important for 52.7% to 74.2% of younger doctors and 56.4% to 73.2% of older doctors. Among respondents aged 45 and over, this group of motives also includes the product brand and manufacturer; in the younger group, these factors were relatively less important.

The third group consists of motivators with an average value ranging from 3.0 to 3.4. Individual factors were important in the purchasing decision-making process for 31.2% to 51.6% of younger respondents and for 36.1% to 51.8% of older respondents. These are factors related to marketing activities (sales promotions at the point of sale, display at the point of sale), the income level of buyers, curiosity about new products and interest in organic/bio products. In the category of doctors aged 45 and over, the appearance of the packaging was also included in this group of factors.

The weakest motivating factors in relation to purchasing decisions for dairy products in both age groups of doctors were in-store tastings, loyalty programmes and product trends. The arithmetic mean of these factors was lower than 3.0 and they were important for less than one third of the doctors surveyed. The appearance of the packaging was also not an important factor for the younger respondents in this group.

When comparing the ratings of individual purchasing determinants by age (Table 1), it was noted that respondents aged 27–44 placed relatively greater importance than older respondents (45 and over) on five factors, with the largest differences concerning sales promotions at the point of sale (the difference in mean values was 0.21), as well as health properties (0.13) and nutritional value of products (0.13). In addition, it was noted that respondents aged 45 and over rated the importance of twenty factors higher than younger doctors, with the greatest differences concerning product brand (the difference in average values was 0.42), expiry date (0.34), manufacturer (0.31), product fashion (0.29), quality certificates (0.24) and tasting at the point of sale (0.21).

In the next step, an attempt was made to answer the question of whether there is a statistically significant difference between doctors aged up to 45 and those aged over 45 in their assessment of the motivational strength of these factors. For this purpose, the Mann–Whitney U test was performed (Table 2).

The non-parametric Mann–Whitney U test, at a significance level of *p* ≤ 0.05, showed statistically significant differences between the categories of doctors aged up to 44 and over 45 in the assessment of the motivational strength of only 3 out of 26 motivators (use-by date, manufacturer, product brand) taken into account in the purchasing decision-making process in the dairy products market. In these cases, the average rank was higher for older respondents.

In summary, our analysis of the arithmetic means shows that the hierarchy of motivators influencing the purchase of dairy products differs between the distinguished age groups of doctors. Although product composition ranked first in both groups, indicating its key role in the purchasing decision-making process, the arrangement and strength of the other motivators show some variation (younger respondents rated 5 out of 26 motivators higher; older respondents rated 20 out of 26 motivators higher). Younger respondents rate in-store promotions, health benefits, nutritional value, curiosity about new products and in-store display higher, which means that they attach greater importance to the impact of in-store marketing activities and the health attributes of dairy products. Older respondents, on the other hand, place greater importance on product brand, expiry date, manufacturer, product design, quality certification and the opportunity to sample products at the point of sale. This suggests that elements related to trust, safety and consumer trends are more important to them. The differences in ratings are also confirmed by the Mann–Whitney U test, which showed statistically significant differences (*p* < 0.05) in the assessment of 3 out of 26 variables (use-by date, manufacturer, product brand) that were more important for older respondents than younger ones. Therefore, research hypothesis H1, which states that there are age-related differences in the importance of individual motivators in purchasing decisions, is true. The validity of research hypothesis H1 is confirmed by the existence of certain differences in the hierarchy and strength of motivators between the analysed groups of respondents, as well as the fact that the Mann–Whitney U test showed a statistically significant difference in the case of three variables. In all these cases, the average rank for the older category of respondents is higher than the average rank for the younger category of respondents, which indicates that these motivators are more important for older doctors than for younger ones.

### 3.2. Hidden Factors Influencing Purchasing Decisions

To determine the optimal number of latent variables that explain the correlation structures between observable variables (such as ‘factors influencing purchasing decisions for dairy products’), exploratory factor analysis (EFA) was conducted for all respondents, as well as for categories distinguished based on the age of the surveyed doctors. Principal component analysis was used to identify the main factors. Before proceeding with the analysis, the data was standardised.

In the first step, Cronbach’s alpha coefficient was calculated to assess the reliability of the measurement tool. The analysed catalogue of 26 variables relating to purchasing motives achieved an alpha coefficient of 0.925 for all respondents, 0.900 for the younger category of doctors and 0.926 for the older category, which indicates a very high reliability of the scale and testifies to its strong internal consistency.

Next, an exploratory factor analysis was performed using the principal component method for all respondents and for categories separated by age. This resulted in the identification of five latent factors (principal components). For the entire sample, as well as for the age groups below and above 45, the number of factors was determined using the Kaiser criterion, according to which only those factors with an eigenvalue greater than 1 were included.

In each case, these factors explain over 61% of the cumulative eigenvalues (Table 3). Although the cumulative percentage of own values at 60% is not very high, it should be emphasised that in social research, which includes phenomena and processes with high variability and multidimensionality, it is considered acceptable and sufficient to identify hidden factors that can be interpreted both theoretically and practically [55]. The relatively lower cumulative percentage may result from the fact that in the analysed group, despite similarities in education and occupation, there were also significant individual differences affecting the dispersion of responses and, thus, the level of explained variance obtained.

The explained variance was highest in the category of respondents over 45 years of age (66%); i.e., in this group, the factors explained the largest part of the total variability (these factors best captured the hidden relationships between the variables). The KMO (Kaiser–Meyer–Olkin) index, known as a measure of the adequacy of variable selection, exceeded the recommended threshold of 0.7 for all analysed categories, which indicates the validity of factor analysis. In all the categories we identified, Bartlett’s sphericity test proved to be statistically significant (*p* < 0.001), confirming the existence of a relationship between the variables and the validity of the analysis.

When analysing the internal structure of individual factors (Table 4), clear differences were observed between the group of respondents under and over 45 years of age, as well as between each of these categories and the total number of respondents. Each factor represents a group of characteristics that jointly explain part of the variability in responses, and high factor loadings (>0.5) indicate which variables are dominant in a given factor.

Interpreting the main components in terms of consumer profiles identified on the basis of factors determining purchasing decisions in the dairy product market, five profiles/segments were identified for each category of respondents analysed (Table 5).

The first principal component for all categories of respondents indicates health motivation—it shows that consumers make purchasing decisions based primarily on the health benefits, quality and natural character of dairy products. Although the hierarchy of individual variables differs slightly depending on age, the structure of the factor remains stable, confirming its universal nature.

The second principal component, for all respondents and for both age categories, despite differences in the hierarchies of individual variables, is also universal and can be described as the marketing attractiveness of the product—it indicates that decisions are made under the influence of consumer trends created by marketers, marketing activities undertaken at the point of sale and the aesthetics of the packaging.

The third principal component is characterised by significant differences in purchasing motivations in the distinct categories of respondents. The result of the factor analysis therefore indicates a diverse structure of this factor depending on the group analysed. In the case of all respondents and those aged 45 and over, purchasing decisions are largely shaped by established consumer habits and family preferences. Despite the indicated similarity, the structure of the factor differs between these categories: among all respondents, purchasing decisions are additionally motivated by sensory properties and openness to new products (habits and consumer openness), while among older consumers, package size is an important determinant. In the case of people under 44, the factor reflects a strong focus on the pragmatic and economic aspects of purchasing dairy products. In this group, the highest factor loadings were obtained for variables related to price and package size, which indicates rational purchasing decisions based on the perceived value of the product. In the younger group of respondents, the expiry date and country of origin of the product were also important, suggesting growing health awareness and interest in the quality and origin of food.

The fourth principal component shows a clear difference in structure depending on the category of respondents analysed. The factor analysis results indicate that for the majority of respondents, economic aspects (price, income level) play a dominant role, but so do pragmatic aspects (packaging size, availability in stores). This pattern suggests that consumer decisions are largely influenced by economic and pragmatic motivations related to the assessment of the cost-effectiveness of a purchase and the availability of a product. In contrast, among people under 44, this factor indicates routine motivations, as evidenced by high variable loads: habits, availability in stores, sensory properties and family preferences. Among people aged 45 and over, this factor indicates sensory attractiveness, as evidenced by high loads of the variables such as sensory properties, curiosity about new products and expiry date.

A clear differentiation in the structure of the main component depending on the category analysed is also evident in the case of the fifth principal component. For the general population and younger respondents, the fifth factor indicates a strong attachment to food safety issues, as evidenced by high factor loadings of variables: expiry date, product brand, manufacturer and country of origin. The fifth factor takes on a different structure in the group of people over 45, where economic rationality plays a decisive role. This factor consists of the variables price, income level and availability in stores.

The diversity of the structure of latent factors identified through exploratory factor analysis using the principal component method indicates significant differences in the determinants of respondents’ purchasing decisions depending on their age, which justifies the distinction of different consumer profiles for dairy products and confirms the second research hypothesis (H2). These differences are important for the design of marketing and communication strategies in the dairy product market. Market segmentation should take into account not only age but also the dominant decision-making motivations (Table 4).

**Figure 2:**
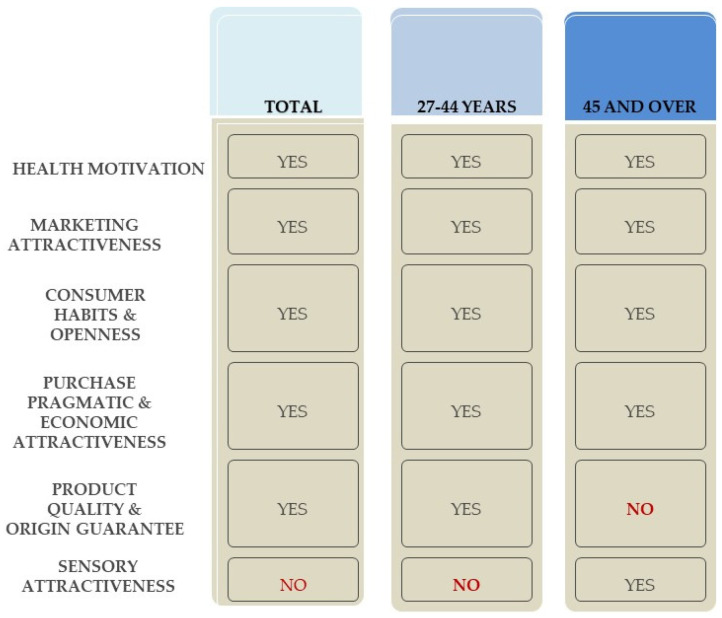
Segments of dairy product consumers—total respondents and age categories. Source: own study.

To provide a more comprehensive presentation of the factor analysis results, in addition to the detailed data shown in Table 5, a graphical summary (Figure 2) has been included, illustrating the five identified consumer segments for both the total respondents and the individual age categories.

## 4. Discussion

According to the authors of this article, it is difficult to directly relate the results of this empirical research to the results of other authors’ studies. This is due to the aforementioned research gap, which particularly concerns the respondents of the study, i.e., a specific professional group of doctors, as well as its selected demographic characteristics, i.e., age. Furthermore, the originality of this study’s results is also determined by its objective, which was to identify the factors influencing the purchasing decisions of Polish doctors on the dairy product market, taking into account the age of the respondents, as well as to propose a segmentation of respondents with a similar factor profile in both groups of doctors surveyed in comparison to the total number of respondents. However, the obtained results can be related to the results of studies by other authors dealing with factors determining purchasing behaviour in the food market or in the context of the product group under study (i.e., dairy products). It is worth adding here that there are many more scientific studies on various approaches to factors influencing the purchasing behaviour of final buyers in the context of different consumer product groups. A good example of this is the research carried out by Liczmańska [56]. The results of her research indicate that the three key factors determining consumer behaviour are product quality, price and product appearance, with price being treated as the market’s assessment of the product and its impact on purchasing decisions depending on a combination of product characteristics and quality level. It can therefore be concluded that these results correspond to some extent with the results presented in the article, where the most important purchasing motives in the market for the analysed products were factors related to quality and health. The quality factor, alongside price, was also one of the key determinants of product purchases when the research sample consisted of young buyers representing Generation Z [57]. In this case, the importance of the price factor may be related to the fact that Generation Z is represented by relatively young people who are at the beginning of their professional careers and therefore have limited income or are still in education [58].

When identifying purchasing determinants, it is worth noting that, especially in the context of food, quality is a complex and graded concept encompassing many aspects to which consumers pay attention. These include, for example, sensory and health characteristics, shelf life and convenience in preparation for consumption [59]. Other authors share this view, stating that, depending on the type of food product and the type of buyer, quality may consist of sensory, functional, economic, nutritional and health indicators, as well as consumer preferences [60]. It is also worth adding that some of these characteristics often cannot be assessed at the time of purchase but only when the food is being prepared for consumption or after it has been consumed. However, quality in its broadest sense is considered a key factor influencing purchasing behaviour, whose importance increases with growing awareness of nutrition [59,61]. It can therefore be assumed that in the case of the present study, both the professional group and the age of the respondents indicated a relatively high level of awareness in the area of conscious choice of various food products, including dairy products. The aspect of health as an important determinant of purchasing is also repeated in the results of other studies on consumer behaviour in the food market. A good example of this is the study conducted by [62] Wojciechowska-Solis on consumers’ interest in local food. Consumers decide to buy local food for many reasons, primarily because of its higher nutritional value or associated health benefits. An interesting study on changes in the importance of factors determining consumer purchasing decisions in 2007–2022 was conducted by [27] Antošová and Stávková. Monitoring the importance of factors related to marketing tools (such as product parameters, price, discounts, product quality, brand, advertising, the opportunity to try something new, recommendations from friends and family, expert recommendations, latest trends, design, habits and traditions, necessity and previous experience) showed that for all monitored factors, there were only minor changes in consumers’ perceptions of their importance over 15 years. At the same time, it was shown that under the influence of current trends and changes in society, new factors influencing consumers have emerged, especially those based on sustainable development and health awareness trends. In the context of these results, it can be concluded that the research results presented in this study correspond to these trends, taking into account the health aspects of dairy products and, above all, referring to the opinion-forming role of the medical community.

This study confirmed the existence of significant differences in the factors influencing respondents’ purchasing decisions depending on their age. Ogundijo et al. [32] showed that age was an important characteristic that influenced the factors determining food purchasing decisions in the academic community. They also emphasised that a detailed study of specific factors influencing personal preferences in food purchasing can help to understand complex decision-making processes and promote healthy eating habits. The study by Anisimova and Vrontis [63] also shows that age is an important characteristic of buyers that determines their deep trust in organic food. This confirms that the age of consumers is an important determinant of food purchasing decisions. These differences point to the need for market segmentation and the adaptation of marketing strategies and food policy to the specific needs of different age groups.

## 5. Conclusions

The achievement of the research objective has allowed new insights to be added to the literature on purchasing decisions for dairy products and the segmentation of buyers of these products, taking into account the age of the respondents. In addition, conducting a study with a group of doctors perceived as experts fills a research gap in the literature and makes an important contribution to the discussion on the factors shaping the purchasing behaviour of doctors as buyers. The results of this study on the hierarchy of factors influencing the purchasing decisions of the distinguished age groups of doctors, i.e., those aged 27–44 and 45 and over, compared to the total number of respondents, are of great importance. Although product composition ranked first in all groups, indicating its key role in the purchasing decision-making process, the hierarchy and strength of other motivators show some variation. Younger respondents attach greater importance to the influence of marketing activities at the point of sale and the health attributes of dairy products. Older respondents value aspects related to trust and safety as well as consumer trends more highly. Therefore, research hypothesis H1, which states that the importance of individual motivators in purchasing decisions varies depending on the age of the doctors (respondents), proved to be true. This is an important indication for companies in the dairy sector in terms of market segmentation, designing marketing strategies for dairy products (including, among others, the launch of new products) and adapting the product range to different target groups. In addition, the results of this study may provide important guidance for decision-makers from institutions influencing public health in the development of campaigns aimed at promoting healthy eating habits that include the consumption of dairy products.

Valuable guidance for both dairy product manufacturers and representatives of institutions involved in nutrition issues can also be derived from the identification of hidden factors influencing purchasing decisions in the dairy product market among doctors aged up to 44 and over 45, compared to the overall respondents. Exploratory factor analysis using the principal component method reveals that the structure of hidden factors shaping purchasing decisions differs significantly depending on the age of the respondents, which confirms the research hypothesis (H2). Although the first hidden factor (health motivation, quality and natural character of products) and the second hidden factor (marketing attractiveness of the product) are common to both age groups, there are significant differences in the composition and hierarchy of the variables that make up the other factors. These differences indicate individualised conditions for making purchasing decisions depending on the age of the respondents. These differences indicate individualised determinants of purchasing decisions depending on the age of the respondents.

In the last step of this study, consumer segments were identified based on the similarity of factor profiles in the general population and among respondents aged up to 44 and over 45. The analysis indicates the existence of both universal and diverse purchasing motivations, which necessitates a different focus for marketing activities.

The dominant segment, present in both age groups, are buyers motivated by health considerations. For this segment, an effective marketing strategy should be based on emphasising the health benefits, natural composition and high quality of dairy products. Communication should highlight the role of dairy products in a healthy diet and should include educational elements on the nutritional value and food safety of this product group.

The second universal segment are consumers who are attracted by the marketing appeal of products. In this case, marketing activities should focus on the aesthetics of packaging, appropriate display at points of sale, attractive promotions and the systematic introduction of new products. This strategy allows you to attract the attention of both younger and older consumers who are susceptible to market trends.

Another segment consists of buyers who are guided by the economic and pragmatic attractiveness of purchases. In both age groups, the favourable price–quality ratio should be emphasised, but the differentiation concerns specific communication accents. In the case of younger consumers, the strategy should take into account the relationship between price and package size, shelf life and product origin. For older buyers, it is important to highlight the relationship between price and income level and the availability of the product in stores, which increases the sense of rationality of the choice.

An important segment in both age groups is also made up of consumers guided by shopping habits and customs. In the younger age group, these decisions are strongly linked to individual and family taste preferences and habits regarding where to shop, which suggests the need to refer to family values and the continuation of proven eating traditions and easy availability in stores in promotional messages. In the older age group, attachment to specific forms of packaging is more important, so marketing strategies should emphasise stability, convenience and repeatability of shopping experiences.

Among younger respondents, a segment of consumers who pay particular attention to product quality and origin was also identified. An effective strategy for this segment should emphasise the manufacturer’s reputation and brand recognition, which increases trust and loyalty to the product. In contrast, among older buyers, a segment of consumers guided by the sensory appeal of products emerged. In this case, marketing activities should focus on taste and aroma properties, offering new variants and organising tastings, as well as emphasising freshness and shelf life.

In summary, marketing strategies in the dairy market should combine universal health and aesthetic messages with communication accents tailored to the age of consumers. Younger groups require the rationality of purchase, quality and origin of products to be highlighted, while older groups prefer to emphasise tradition, stability and sensory appeal. Such a differentiated approach allows promotional activities to be better tailored to the specific characteristics of individual buyer segments.

The results of this study are important for professionals dealing with public nutrition issues, as well as for marketers of dairy companies responsible for developing effective marketing strategies. These results can be used by relevant institutions in public health campaigns, as they emphasise the importance of promoting regular consumption of dairy products, and their key advantage is that they come from doctors, who are considered one of the most trusted professional groups and whose opinions can influence the purchasing decisions of various consumer groups and promote healthy eating habits. These results can provide important arguments for those developing strategies and educational campaigns aimed at various social groups.

The significant limitations of this study include the unrepresentative nature of the research sample. Further research could focus on identifying the factors influencing the purchasing decisions of doctors as individual buyers in the dairy product market, taking into account representative samples in Poland and other countries, including EU Member States. In addition, it is worth considering a comparative analysis of the purchasing motivations of this group with the consumer attitudes of people without medical education.

## Figures and Tables

**Table 1:** Motives for purchasing decisions in the dairy product market and age categories.

Variables	27–44 Years									45 and Over									Difference in Averages (27–44)—(45 and over)	Rank for the Total
	Response Rate (%)					$\bar{X}$	Rank	SD	V	Response Rate (%)					$\bar{X}$	Rank	SD	V		
	1	2	3	4	5					1	2	3	4	5						
product composition	0	6.5	14	24	56	4.29	1	0.939	21.9	0.9	4.6	10	33	51	4.29	1	0.897	20.9	0.00	1
health values	0	2.2	18	30	50	4.27	2	0.836	19.6	1.9	6.5	11	37	44	4.14	5	0.981	23.7	0.13	2
no preservatives	0	6.5	18	28	47	4.16	4	0.947	22.8	2.8	4.6	10	33	49	4.21	2	0.996	23.7	−0.05	3
nutritional values	0	4.3	16	32	47	4.23	3	0.874	20.7	1.9	4.6	17	35	42	4.10	6	0.966	23.6	0.13	4
sensory properties (taste, smell)	1.1	5.4	16	45	32	4.02	5	0.897	22.3	2.8	3.7	14	31	49	4.19	3	1.000	23.9	−0.17	5
best before date	2.2	8.6	26	32	31	3.82	7	1.042	27.3	2.8	1.9	18	32	45	4.16	4	0.968	23.3	−0.34	6
availability in store	1.1	3.2	23	44	29	3.97	6	0.865	21.8	1.9	5.6	20	41	32	3.94	7	0.955	24.2	0.03	7
habit	3.2	5.4	17	58	16	3.78	8	0.895	23.7	3.7	3.7	20	41	32	3.93	8	1.002	25.5	−0.15	8
quality certificate	5.4	5.4	31	33	25	3.67	12	1.077	29.3	3.7	6.5	20	34	35	3.91	9	1.072	27.4	−0.24	9
country of origin of the product	3.2	15	19	31	31	3.72	9	1.155	31.0	4.6	8.3	20	36	31	3.80	11	1.109	29.2	−0.08	10
family preferences	2.2	12	23	42	22	3.69	10	1.011	27.4	4.6	4.6	18	52	21	3.81	10	0.981	25.7	−0.12	11
local product	2.2	11	25	42	20	3.68	11	0.991	26.9	4.6	11	12	45	27	3.79	12	1.103	29.1	−0.11	12
traditional recipes	3.2	11	29	37	20	3.60	13	1.034	28.7	3.7	7.4	22	44	23	3.75	13	1.015	27.1	−0.15	13
price	2.2	8.6	36	34	19	3.60	14	0.968	26.9	4.6	7.4	25	38	25	3.71	14	1.068	28.8	−0.11	14
package size	2.2	14	31	36	17	3.52	15	1.007	28.6	4.6	9.3	30	41	16	3.54	17	1.018	28.8	−0.02	15
product brand	5.4	13	42	32	7.5	3.24	21	0.960	29.6	5.6	6.5	25	44	20	3.66	15	1.052	28.7	−0.42	16
manufacturer	3.2	16	44	26	11	3.25	20	0.963	29.6	4.6	8.3	29	43	16	3.56	16	1.007	28.3	−0.31	17
curiosity about a new product	3.2	15	31	36	15	3.44	16	1.026	29.8	7.4	14	29	32	19	3.40	20	1.160	34.1	0.04	18
organic/bio products	6.5	20	22	33	18	3.37	17	1.187	35.2	6.5	15	27	33	19	3.43	19	1.146	33.4	−0.06	19
income level	8.6	13	39	24	16	3.26	19	1.141	35.0	8.3	11	29	32	19	3.44	18	1.170	34.0	−0.18	20
sales promotions at point of sale	8.6	19	26	30	16	3.26	18	1.197	36.7	11	20	32	25	11	3.05	22	1.163	38.1	0.21	21
display at point of sale	11	17	41	22	9.7	3.02	22	1.103	36.5	14	14	36	28	8.3	3.03	23	1.148	37.9	−0.01	22
packaging appearance	13	20	37	23	7.5	2.91	23	1.120	38.5	9.3	19	30	35	6.5	3.10	21	1.085	35.0	−0.19	23
loyalty programmes	16	18	42	19	4.3	2.77	24	1.075	38.8	14	22	33	24	6.5	2.87	24	1.128	39.3	−0.10	24
tasting at point of sale	29	25	19	19	7.5	2.52	25	1.299	51.5	20	25	26	19	10	2.73	25	1.265	46.3	−0.21	25
trend products	29	25	31	11	4.3	2.37	26	1.140	48.1	24	19	32	15	9.3	2.66	26	1.254	47.1	−0.29	26

Symbols: $\bar{X}$—arithmetic mean—represents the mean score for each variable; SD—standard deviation—showing the dispersion of responses around the mean; V—coefficient of variation—represents the coefficient of variation, calculated as SD divided by the mean, then multiplying the result by 100, providing a standardised measure of variability. Rank indicates the relative importance of the variable based on mean values. Difference in averages (27–44)—(45 and over) shows the difference in mean scores between the two age groups. Rating scale: 1—completely unimportant, 2—not very important, 3—difficult to say, 4—rather important, 5—very important. Source: own research results.

**Table 2:** Analysis of the significance of differences between respondents’ answers by age category.

Variables	Age	Mean Rank	Sum of Ranks	Mann–Whitney U	Asymptotic Significance (Two-Tailed)
health values	27–44	104.00	9672.00	4743.0	0.464
	45 and over	98.42	10,629.00		
nutritional values	27–44	104.42	9711.00	4704.0	0.407
	45 and over	98.06	10,590.00		
best before date	27–44	90.54	8420.50	4049.5	0.013
	45 and over	110.00	11,880.50		
product composition	27–44	102.12	9497.50	4917.5	0.779
	45 and over	100.03	10,803.50		
sensory properties (taste, smell)	27–44	93.30	8677.00	4306.0	0.063
	45 and over	107.63	11,624.00		
no preservatives	27–44	98.73	9181.50	4810.5	0.578
	45 and over	102.96	11,119.50		
organic/bio products	27–44	99.60	9263.00	4892.0	0.744
	45 and over	102.20	11,038.00		
quality certificate	27–44	93.55	8700.00	4329.0	0.078
	45 and over	107.42	11,601.00		
traditional recipes	27–44	96.16	8942.50	4571.5	0.250
	45 and over	105.17	11,358.50		
manufacturer	27–44	89.87	8357.50	3986.5	0.008
	45 and over	110.59	11,943.50		
country of origin of the product	27–44	99.19	9224.50	4853.5	0.670
	45 and over	102.56	11,076.50		
local product	27–44	96.01	8928.50	4557.5	0.233
	45 and over	105.30	11,372.50		
availability in store	27–44	100.81	9375.00	5004.0	0.963
	45 and over	101.17	10,926.00		
tastings at the point of sale	27–44	95.72	8902.00	4531.0	0.221
	45 and over	105.55	11,399.00		
loyalty programmes	27–44	98.58	9167.50	4796.5	0.569
	45 and over	103.09	11,133.50		
display at point of sale	27–44	99.98	9298.50	4927.5	0.811
	45 and over	101.88	11,002.50		
sales promotions at the point of sale	27–44	106.55	9909.00	4506.0	0.196
	45 and over	96.22	10,392.00		
price	27–44	96.34	8959.50	4588.5	0.270
	45 and over	105.01	11,341.50		
product brand	27–44	87.12	8102.50	3731.5	0.001
	45 and over	112.95	12,198.50		
packaging appearance	27–44	95.40	8872.00	4501.0	0.189
	45 and over	105.82	11,429.00		
package size	27–44	99.72	9274.00	4903.0	0.762
	45 and over	102.10	11,027.00		
income level	27–44	95.62	8892.50	4521.5	0.207
	45 and over	105.63	11,408.50		
product trend	27–44	94.02	8744.00	4373.0	0.103
	45 and over	107.01	11,557.00		
habits	27–44	95.53	8884.00	4513.0	0.183
	45 and over	105.71	11,417.00		
curiosity about new products	27–44	101.41	9431.50	4983.5	0.923
	45 and over	100.64	10,869.50		
family preferences	27–44	97.00	9021.00	4650.0	0.334
	45 and over	104.44	11,280.00		

Source: own research results.

**Table 3:** Hierarchy of factors according to their eigenvalues determined using the Kaiser criterion for all respondents and age groups below and above 45 years of age.

Main Factor	Eigenvalue			Cumulative Eigenvalue			% of Total Eigenvalues (Variance)			Cumulative % of Eigenvalues		
	Total	27–44 Years	45 and Over	Total	27–44 Years	45 and Over	Total	27–44 Years	45 and Over	Total	27–44 Years	45 and Over
1	9.175	7.655	10.541	9.175	7.655	10.541	19.923	19.682	20.115	19.923	19.682	20.115
2	3.139	3.712	2.788	12.314	11.367	13.329	16.332	14.780	18.797	36.255	34.461	38.912
3	1.599	2.021	1.541	13.913	13.388	14.870	9.663	10.199	9.534	45.918	44.661	48.447
4	1.283	1.481	1.249	15.196	14.869	16.119	9.198	9.914	9.050	55.116	54.575	57.497
5	1.037	1.221	1.120	16.233	16.090	17.239	7.321	7.315	8.812	62.437	61.890	66.309

For “age 27–44”—KMO = 0.802, Bartlett’s sphericity test is significant, chi2 = 1219.391 and *p* < 0.001; for “45 and over”—KMO = 0.8742, Bartlett’s sphericity test is significant, chi2 = 1747.251 and *p* < 0.001. Source: own study.

**Table 4:** Results of factor analysis of determinants of purchasing decisions in the dairy product market (for young and older respondents).

Variables	Factors									
	1		2		3		4		5	
	27–44 Years	45 and Over	27–44 Years	45 and Over	27–44 Years	45 and Over	27–44 Years	45 and Over	27–44 Years	45 and Over
no preservatives	0.806	0.83	0.034	−0.012	0.138	0.083	0.094	0.16	0.006	0.175
health values	0.827	0.709	−0.021	0.026	0.145	0.322	0.137	0.15	−0.05	0.143
organic/bio products	0.76	0.725	0.216	0.311	−0.089	0.079	0.001	0.002	−0.1	0.03
quality certificate	0.73	0.737	0.137	0.264	0.006	0.159	0.148	−0.012	0.132	0.201
product composition	0.694	0.75	−0.193	−0.063	0.449	−0.081	0.183	0.331	0.062	0.124
traditional recipes	0.704	0.575	0.199	0.227	−0.145	0.046	−0.003	0.271	0.155	0.396
local product	0.663	0.644	0.038	0.292	0.125	−0.151	0.047	0.266	0.246	0.2
nutritional values	0.685	0.672	−0.18	0.077	0.291	0.409	0.176	0.144	0.244	−0.078
product trend	0.02	0.035	0.734	0.823	0.01	0.065	−0.117	0.115	0.157	0.076
tastings at point of sale	0.215	0.257	0.774	0.685	−0.143	0.075	0.082	0.195	0.025	0.13
exposure at point of sale	0.03	0.067	0.662	0.72	0.077	0.096	0.342	0.25	0.274	0.319
packaging appearance	−0.133	0.154	0.526	0.758	0.108	0.101	0.181	0.241	0.442	0.117
loyalty programmes	0.018	0.21	0.672	0.642	0.151	0.261	0.159	−0.211	0.14	0.361
sales promotions at point of sale	0.006	0.145	0.678	0.73	0.254	0.268	0.136	0.129	−0.216	0.18
habits	0.019	−0.136	0.108	0.302	−0.073	0.542	0.818	0.417	0.089	0.121
family preferences	0.102	0.176	0.233	0.216	0.347	0.734	0.524	0.243	0.236	0.087
sensory properties (taste, smell)	0.345	0.309	0.144	0.278	0.281	0.254	0.557	0.683	0.247	0.096
curiosity about new products	0.29	0.098	0.45	0.473	0.051	0.112	0.377	0.594	0.314	0.325
price	−0.014	0.169	0.343	0.34	0.741	0.087	−0.081	0.087	0.059	0.754
package size	0.164	0.218	0.27	0.195	0.628	0.629	0.378	−0.077	−0.158	0.506
income level	0.109	0.234	0.54	0.283	0.346	0.086	0.298	0.348	−0.232	0.598
availability in store	0.282	0.255	0.228	0.06	0.39	0.48	0.605	0.27	−0.046	0.554
best before date	0.078	0.253	−0.038	0.117	0.629	0.196	0.323	0.503	0.17	0.124
product brand	0.141	0.382	0.25	0.56	0.044	0.408	0.358	0.345	0.659	−0.153
manufacturer	0.425	0.365	0.08	0.448	0.124	0.444	0.046	0.392	0.664	0.093
country of origin of the product	0.428	0.597	−0.088	0.43	0.567	0.129	−0.11	−0.114	0.387	0.075

Source: own study.

**Table 5:** Segments of respondents identified according to their motives for purchasing dairy products (for the total population, people aged 27–44 and 45 and over).

**Total**	**HEALTH MOTIVATION** No preservatives (0.819) Health benefits (0.755) Organic/bio product (0.754) Quality certificate (0.724) Product composition (0.720) Traditional recipes (0.707) Local products (0.693) Nutritional values (0.642)	**MARKETING ATTRACTIVENESS** Product trends (0.791) Tasting at the point of sale (0.720) Point-of-sale display (0.709) Packaging appearance (0.702) Loyalty programmes (0.646) In-store promotions (0.625)	**CONSUMER HABITS AND OPENNESS** Habits (0.792) Family members’ preferences (0.595) Sensory characteristics (0.542) Curiosity about new products (0.514)	**PURCHASE PRAGMATIC AND ECONOMIC ATTRACTIVENESS** Price (0.707) Packaging size (0.681) Income level (0.589) Availability in store (0.571)	**PRODUCT QUALITY AND ORIGIN GUARANTEE** Best before date (0.605) Product brand (0.543) Manufacturer (0.512) Country of origin (0.505)
**27–44 years**	**HEALTH MOTIVATION** Health benefits (0.827) No preservatives (0.806) Organic/bio product (0.760) Quality certificate (0.730) Traditional recipes (0.704) Product composition (0.694) Nutritional values (0.685) Local product (0.663)	**MARKETING ATTRACTIVENESS** Tasting at the point of sale (0.744) Product trend (0.734) Sales promotions at the point of sale (0.678) Loyalty programmes (0.672) Point-of-sale display (0.662) Income level (0.540) Packaging appearance (0.526)	**PURCHASE PRAGMATIC AND ECONOMIC ATTRACTIVENESS** Price (0.741) Shelf life (0.629) Packaging size (0.628) Country of origin (0.567)	**CONSUMER HABITS AND OPENNESS** Habits (0.818) Availability in stores (0.605) Sensory properties (taste, smell) (0.557) Family preferences (0.524)	**PRODUCT QUALITY AND ORIGIN GUARANTEE** Manufacturer (0.664) Product brand (0.659)
**45 and over**	**HEALTH MOTIVATION** No preservatives (0.830) Product composition (0.750) Quality certificate (0.737 Organic/bio product (0.725) Health benefits (0.709) Nutritional value (0.672) Local product (0.644) Country of origin (0.597) Traditional recipes (0.575)	**MARKETING ATTRACTIVENESS** Product trendiness (0.823) Appearance of packaging (0.758) Sales promotions at the point of sale (0.730) Point-of-sale display (0.720) Tasting at point of sale (0.685) Loyalty programmes (0.642) Product brand (0.560) Manufacturer (0.448)	**CONSUMER HABITS AND OPENNESS** Family member preferences (0.734) Packaging size (0.629) Habits (0.542)	**SENSORY ATTRACTIVENESS** Sensory properties (0.683) Curiosity about a new product (0.594) Shelf life (0.503)	**PURCHASE PRAGMATIC AND ECONOMIC ATTRACTIVENESS** Price (0.754) Income level (0.598) Availability in stores (0.554)

Source: own study.

## Data Availability

Due to ethical restrictions and participant confidentiality, the data cannot be made publicly available. However, the data from this study is available upon request, for researchers who meet the criteria for access to confidential data.
